# Supplementary material for: Preparation, Properties, and Mechanism of Flame-Retardant Poly(vinyl alcohol) Aerogels Based on the Multi-Directional Freezing Method
Source: Int J Mol Sci. 2022 Dec 14;23(24):15919. doi: 10.3390/ijms232415919 (PMC9784135; doi:10.3390/ijms232415919)
Supplement: Supplementary file 1 [file ijms-23-15919-s001.zip › ijms-2039924-supplementary.pdf]

Supporting information

Table S1 TGA Data of α-cellulose and PCF

Sample	T_{donset}(°C)	T_{dmax}(°C)	Char residues(%)	
			600°C	800°C
primordial a-ZrP	231.65	543.6	90.8	90.33
exfoliated a-ZrP	290.4	345.1	37.5	36.4
M1000	308.41	399.33	3.1	2.25

Table S2. TGA Data of a-ZrP

Sample	T_{donset}(°C)	T_{dmax}(°C)	Char residues(%)	
			600°C	800°C
α-cellulose	212.5	337	15.3	10.6
PCF	183.7	214.2	62.3	58.4

Table S3 TGA Data of PVA/PCF_x/α-ZrP_y aerogels

**Sample**	**T_{donset}(°C)**		**T_{dmax1}(°C)**		**T_{dmax2}(°C)**		**Char residues(%)**			
							**600°C**		**800°C**	
	**N_2**	**Air**	**N_2**	**Air**	**N_2**	**Air**	**N_2**	**Air**	**N_2**	**Air**
PVA	266.7	264.8	302.1	309.1	452.3	496.1	7.0	2.8	3.9	2.8
PVA/α-ZrP_{20}	113.2	257.1	322.6	305.3	391.2	464.3	15.0	8.9	14.5	8.7
PVA/PCF_5/α-ZrP_{15}	115.1	219.6	309.4	269.3	464.2	523.8	19.7	16,4	18.7	8.9
PVA/PCF_{10}/α-ZrP_{10}	101.5	201.9	291.5	273.2	459.4	497.6	26.9	16.8	23.7	9.5
PVA/PC_{15}/α-ZrP_5	102.9	205.7	305.7	304.2	464.8	528.9	24.1	9.2	22.9	9.3

PVA/PCF_{20}	126.5	210.6	269.5	246.7	455.3	500	25.7	21.8	24.2	9.9

Table S4. Formulation of flame-retardant PVA aerogels

Sample	component		
	PVA (wt%)	PCF (wt%)	a-ZrP (wt%)
PVA	100	–	–
PVA/a-ZrP_{20}	80	–	20
PVA/PCF_5/a-ZrP_{15}	80	5	15
PVA/PCF_{10}/a-ZrP_{10}	80	10	10
PVA/PCF_{15}/a-ZrP_5	80	15	5
PVA/PCF_{20}	80	20	–

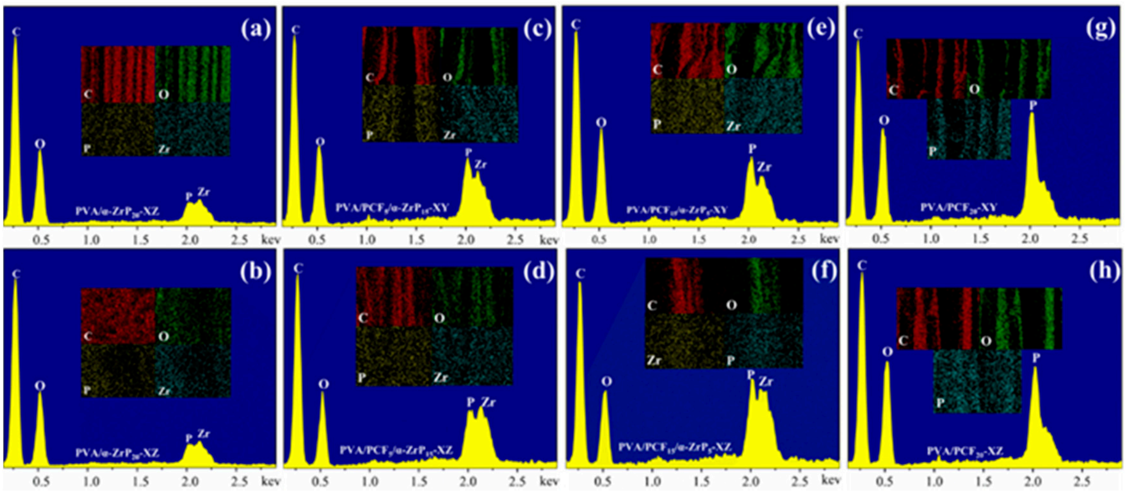

Figure S1. EDS spectra and EDS elemental mapping image for the cross section and longitudinal section of PVA/PCFx/α-ZrP_y: (a) and (b) PVA/α-ZrP_{20}; (c) and (d) PVA/PCF_5/α-ZrP_{15}; (e) and (f) PVA/PCF_{15}/α-ZrP_5; (g) and (h) PVA/PCF_{20}.

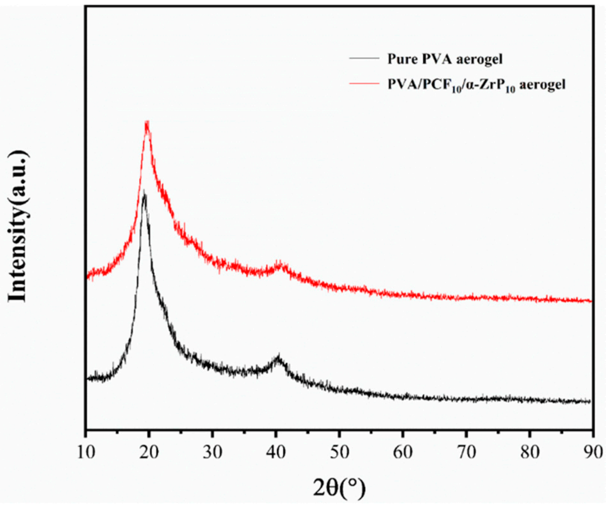

Figure S2. XRD of of PVA aerogel and PVA/PCF$_{10}$/α-ZrP$_{10}$ aerogel

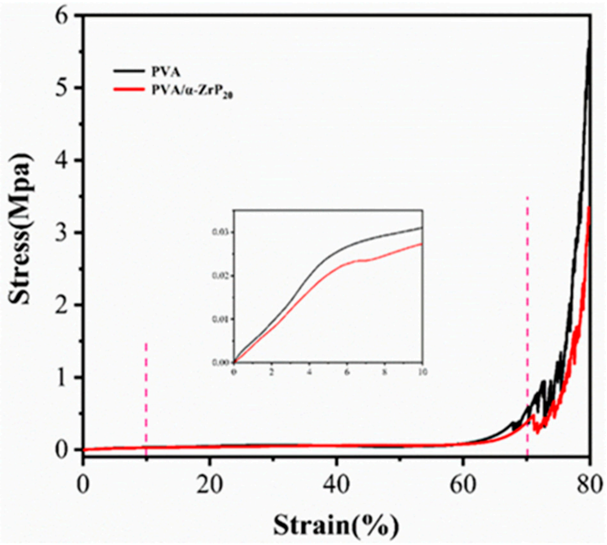

Figure S3. Vertical compressed stress-strain curves of PVA aerogel and

PVA/PCF$_{10}$/α-ZrP$_{10}$ aerogel

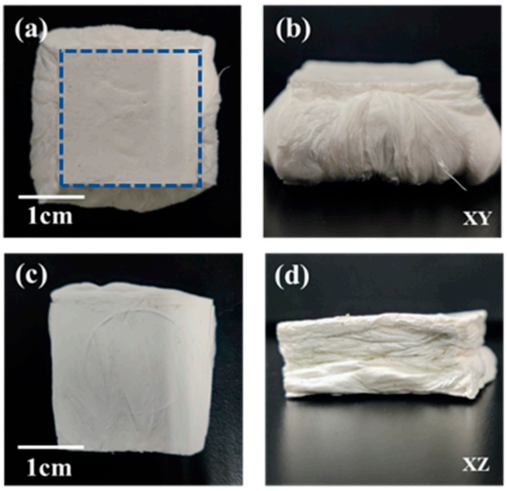

Figure S4. The digital photos of PVA/PCF$_{10}$/α-ZrP$_{10}$ aerogel: (a) top in vertically compressed; (b) flank in vertically compressed; (c) top in horizontally compressed;(d) flank in horizontally compressed.

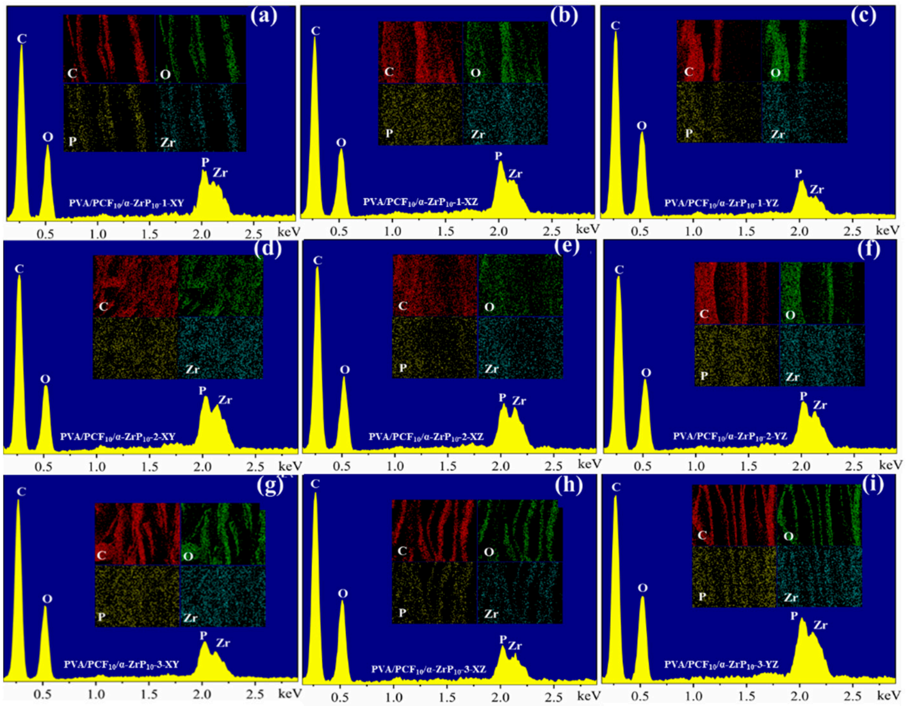

Figure S5. EDS spectra and EDS elemental mapping image for the multi-directional PVA/PCF10/α-ZrP10-x aerogels. (a) PVA/PCF$_{10}$/α-ZrP$_{10}$-1-XY; (b) PVA/PCF$_{10}$/α-ZrP$_{10}$-1-XZ; (C) PVA/PCF$_{10}$/α-ZrP$_{10}$-1-YZ; (d) PVA/PCF$_{10}$/α-ZrP$_{10}$-2-XY; (e) PVA/PCF$_{10}$/α-ZrP$_{10}$-2-XZ; (f) PVA/PCF$_{10}$/α-ZrP$_{10}$-2-YZ; (g) PVA/PCF$_{10}$/α-ZrP$_{10}$-3-

XY; (h) PVA/PCF10/α-ZrP10-3-XZ; (i) PVA/PCF10/α-ZrP10-3-YZ.

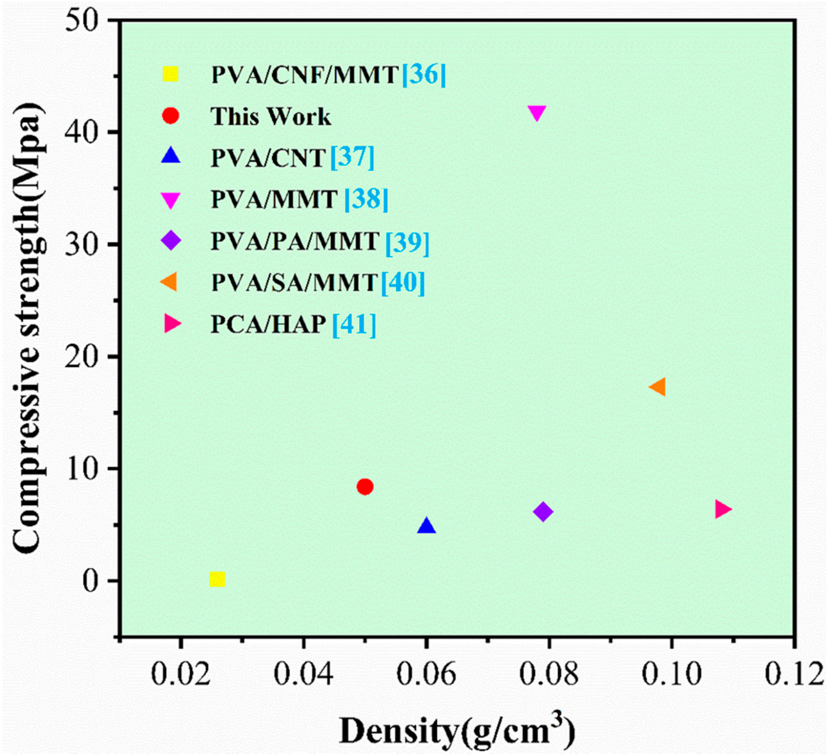

Figure S6. Comparison of this job with other jobs. (References are attached at the end of the page)

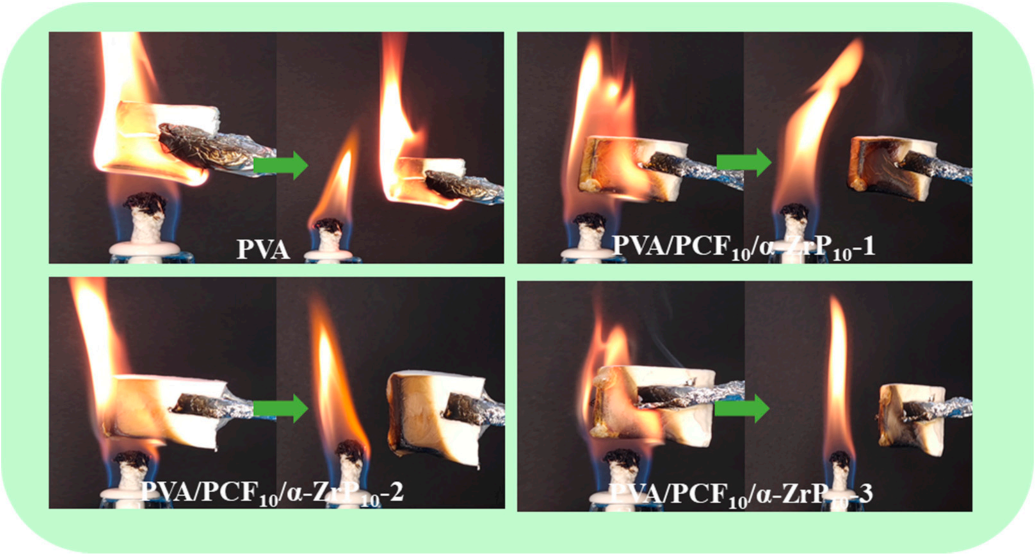

Figure S7. Video screenshot of the combustion for pure PVA, PVA/PCF10/α-ZrP10-1, PVA/PCF10/α-ZrP10-2 and PVA/PCF10/α-ZrP10-3.

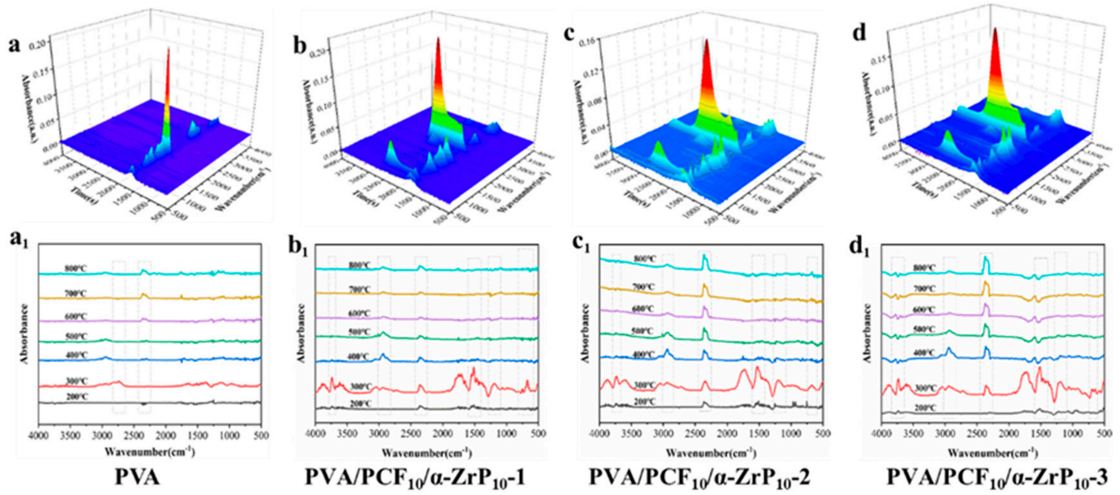

Figure S8 3D TG-FTIR images and FTIR spectra of PVA, PVA/PCF10/α-ZrP10-x.

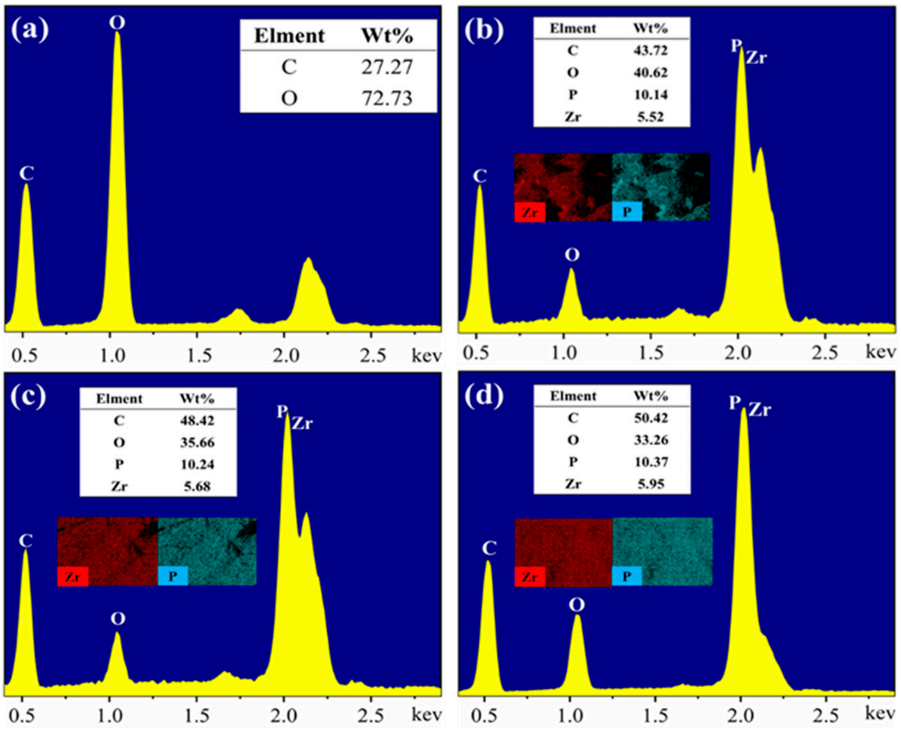

Figure S9. EDX elemental mapping images of the residues of the (a) PVA, (b) PVA/PCF10/α-ZrP10-1, (c) PVA/PCF10/α-ZrP10-2 and (d) PVA/PCF10/α-ZrP10-3 after cone calorimetry.

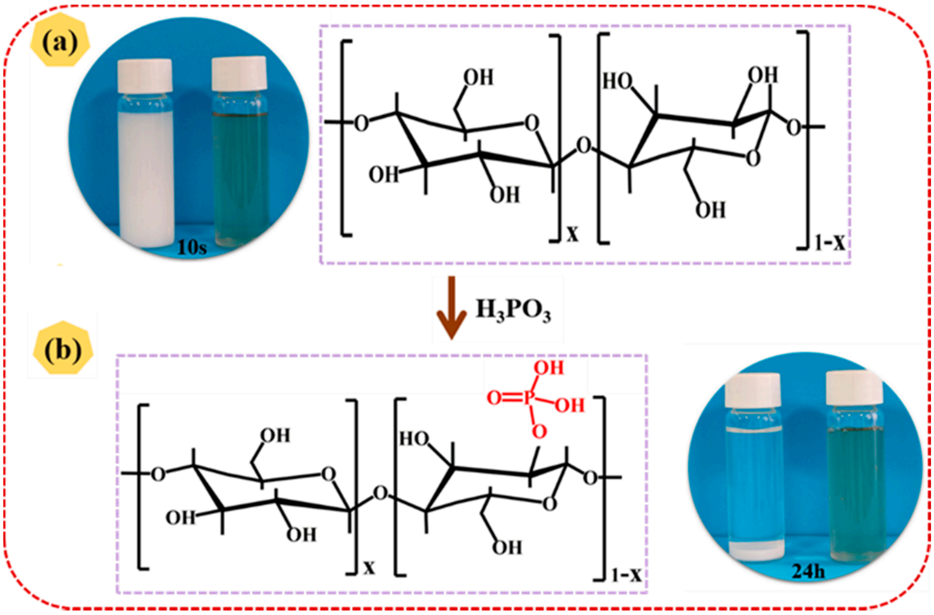

Figure S10. Reaction mechanism for the synthesis of PCF.

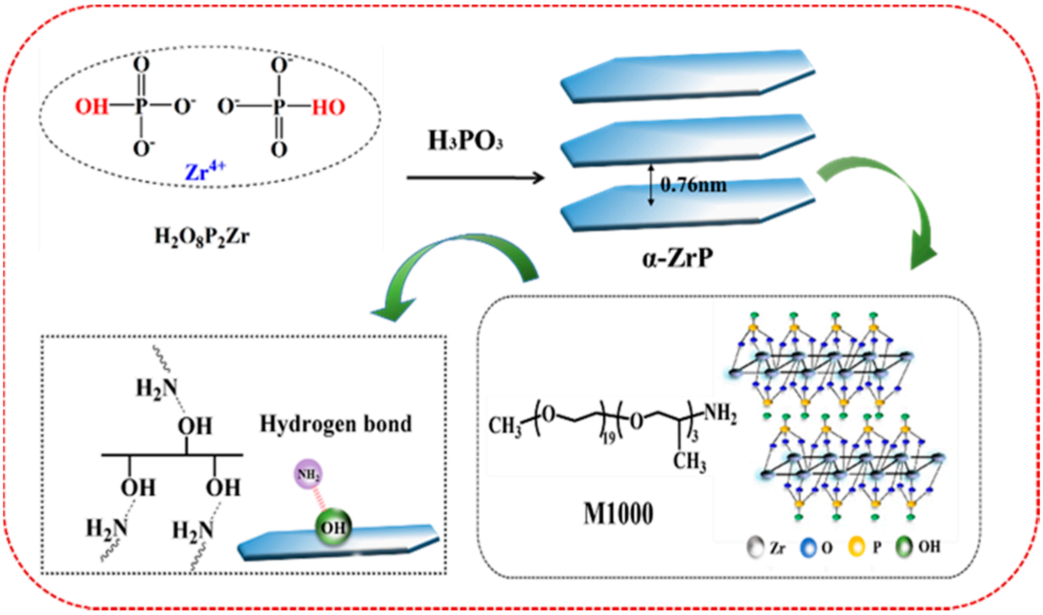

Figure S11. Reaction mechanism for the synthesis of α-ZrP.

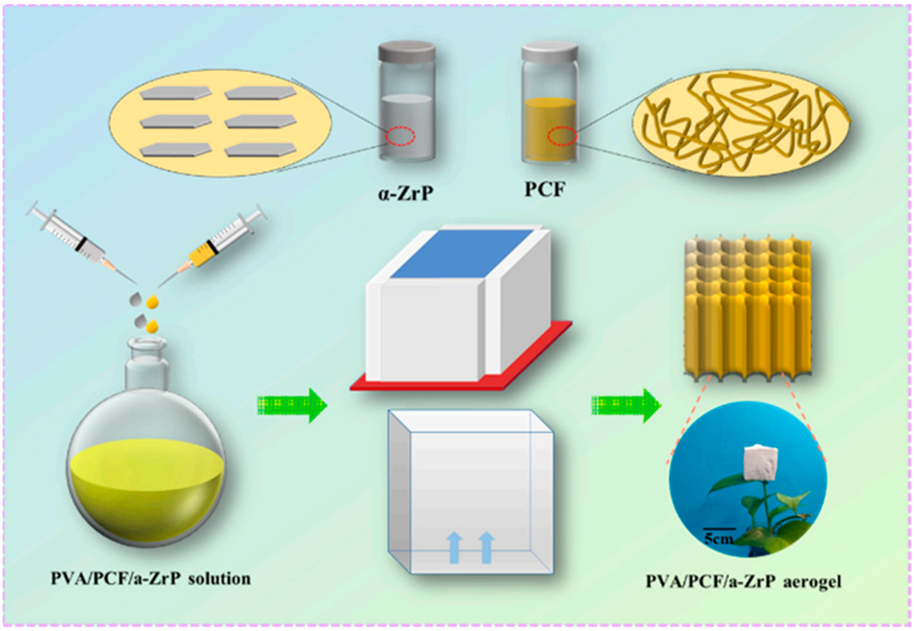

Figure S12. Schematic diagram for the preparation of unidirectional PVA aerogel.

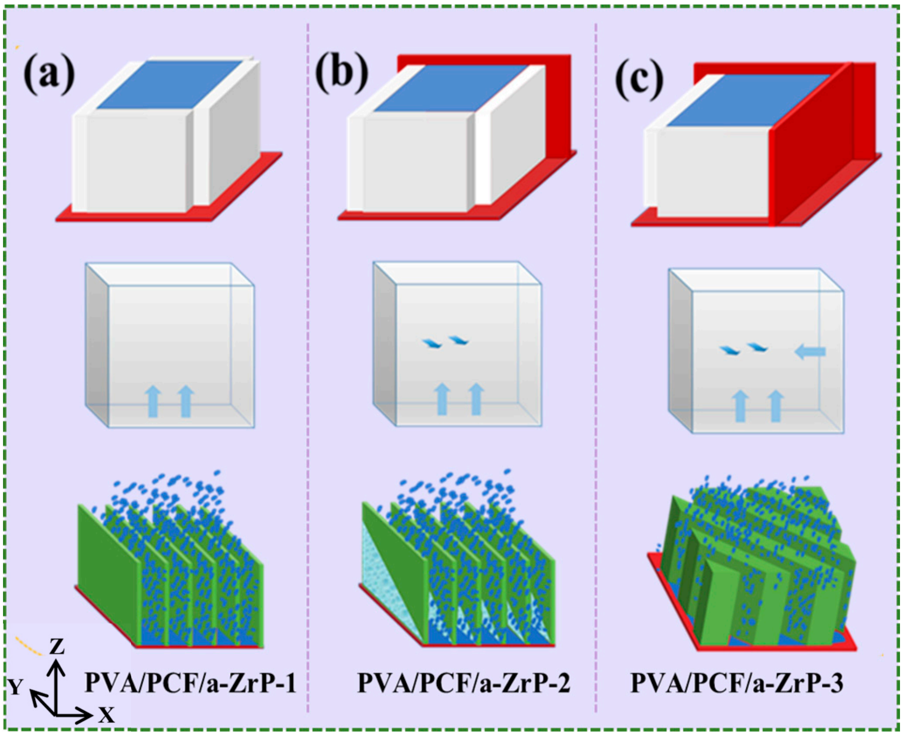

Figure S13. Schematic diagram for the preparation of multi-directional PVA/PCF/a-ZrP-x aerogels
